# Down-regulation of SLC25A20 promotes hepatocellular carcinoma growth and metastasis through suppression of fatty-acid oxidation

**DOI:** 10.1038/s41419-021-03648-1

**Published:** 2021-04-06

**Authors:** Peng Yuan, Jiao Mu, Zijun Wang, Shuaijun Ma, Xiuwei Da, Jian Song, Hongxin Zhang, Le Yang, Jibin Li, Jingyue Yang

**Affiliations:** 1 Department of Pain Treatment, Tangdu Hospital, Air Force Military Medical University, 710038, Xi’an, Shaanxi China; 2 State Key Laboratory of Cancer Biology and Department of Physiology and Pathophysiology, Air Force Military Medical University, 710032, Xi’an, Shaanxi China; 3 Department of Hematology, The First Affiliated Hospital of Chongqing Medical University, 400016, Chongqing, China; 4 Department of Hematology, Xi’an Central Hospital, 710003, Xi’an, Shaanxi China; 5 Battalion of the first Regiment of cadets of Basic Medicine, Air Force Military Medical University, 710032, Xi’an, Shaanxi China; 6 Department of Urology, Xijing Hospital, Air Force Military Medical University, 710032, Xi’an, Shaanxi China; 7 Department of Hepatobiliary Surgery, Xijing Hospital, Air Force Military Medical University, 710032, Xi’an, Shaanxi China; 8 Precision Pharmacy and Drug Development Center, Department of Pharmacy, Tangdu Hospital, Air Force Military Medical University, 710038, Xi’an, Shaanxi China; 9 Department of Oncology, Xijing Hospital, Air Force Military Medical University, 710032, Xi’an, Shaanxi China

**Keywords:** Liver cancer, Liver cancer

## Abstract

Solute carrier family 25 member 20 (SLC25A20) is a mitochondrial-membrane–carrier protein involved in the transport of acylcarnitines into mitochondrial matrix for oxidation. A previous-integrated-proteogenomic study had identified SLC25A20 as one of the top-three prognostic biomarkers in HCC. However, the expression and the biological function of SLC25A20 have not yet been investigated in HCC. In the present study, we found that SLC25A20 expression is frequently down-regulated in HCC cells mainly due to the up-regulation of miR-132-3p. Down-regulation of SLC25A20 is associated with a poor prognosis in patients with HCC. SLC25A20 suppressed HCC growth and metastasis, both in vitro and in vivo, by suppression of G1–S cell transition, epithelial-to-mesenchymal transition (EMT), and induction of cell apoptosis. Mechanistically, SLC25A20 down-regulation promoted HCC growth and metastasis through suppression of fatty-acid oxidation. Altogether, SLC25A20 plays a critical tumor-suppressive role in carcinogenesis of HCC; SLC25A20 may serve as a novel prognostic factor and therapeutic target for patients with HCC.

## Introduction

Hepatocellular carcinoma (HCC) is one of the most common cancers worldwide^1^. Most patients are diagnosed with it in advanced stages due to the lack of specific molecular markers for early detection. In spite of the great progressions that have been made during the recent years, the effective therapies for HCC are still limited, owing to the poor understanding of the molecular mechanisms underlying the metastatic growth of HCC^2,3^. Accordingly, it is critical to identify novel molecular mechanisms involved in the carcinogenesis of HCC, which could provide potential diagnostic and therapeutic strategies in the treatment of this malignance.

The solute carrier family 25 member 20 (SLC25A20), also known as carnitine/acylcarnitine transporter (CACT), is a mitochondrial-membrane–carrier protein involved in the transport of acylcarnitines into mitochondrial matrix for oxidation^4,5^. Previous studies had reported that mutations in SLC25A20 are closely associated with carnitine-acylcarnitine-translocase deficiency, resulting in a variety of metabolic diseases such as hypoglycemia, hepatic dysfunction, and muscle weakness^6–8^. Besides, mutations in SLC25A20 are also usually lethal in the newborns and infants^9^, further implying its critical physiological functions. Recently, a mass spectrometry (MS)-based proteomics study in a large cohort of tumor tissues from 159 HBV-related HCC patients has identified SLC25A20 as one of the robust and representative prognostic proteins in HCC^10^, implying that SLC25A20 may play important roles in the development and progression of HCC. However, the expression and biological functions of SLC25A20 remain largely unexplored in human cancers, especially in HCC.

We conducted the first study on SLC25A20 in HCC with the aim of elucidating its clinical implications, biological functions, and molecular mechanism in this malignancy.

## Materials and methods

### HCC cell lines and HCC tumor tissues sample collection

Six human HCC cell lines (HLF, SNU-354, Huh-7, SNU-368, HLE, and SNU-739) and one hepatocyte (HL-7702) were obtained from the American Type Culture Collection (ATCC) and cultured in Dulbecco’s Modified Eagle’s medium (DMEM) or RPMI-1640 medium supplemented with 10% FBS (Hyclone). All cell lines used in the study have been recently tested by STR DNA profiling and mycoplasma contamination. Additionally, 256 (30 for qRT–PCR; 226 for immunohistochemical analysis) primary HCC tumor tissues were collected at the First Affiliated Hospital of Fourth Military Medical University in Xi’an, China. Written informed consent was obtained from all patients and the tumor tissues were histologically confirmed as hepatocellular carcinoma. The investigation has been approved by the Ethics Committee of the First Affiliated Hospital of Fourth Military Medical University in Xi’an, China.

### Knockdown and forced expression of target genes

For knockdown of SLC25A20 in HCC cells, two siRNAs targeting SLC25A20 were purchased from Genepharma (Shanghai, China). Transfection of siRNAs was carried out with Lipofectamine 3000 (Invitrogen, CA, USA) following the manufacturer’s instructions. The sequences of siRNAs used are given in Supplementary Table S1. For overexpression of SLC25A20 in HCC cells, the coding sequences of SLC25A20 were amplified from cDNA derived from HL-7702 cells and cloned into the pcDNATM3.1(C) vector (Invitrogen, V790-20).

### Real-time PCR and western blot analysis

Total RNA was extracted from HCC cells with an RNA-purification kit (Qiagen, German) and, then, reversely transcribed into cDNAs using a PrimeScript® RT reagent Kit (Takara, Japan). Then, cDNA was processed for real-time PCR with the primers listed in Supplementary Table 2. β-actin was used as an endogenous control and, finally, the relative expression of target genes was calculated with the 2^-ΔΔCt^ method.

For western blot analysis, the cells were lysed in RIPA buffer supplemented with phosphatase-inhibitor cocktail. Protein concentrations in each cell group were determined by the BCA assay (Thermo Scientific Pierce, UK). Protein extracted from each group was electrophoresed on SDS–polyacrylamide gels and, then, transferred onto PVDF membranes. The membranes were blocked with 5% milk, and then incubated with specific primary listed in Supplementary Table 3 and the appropriate horseradish-peroxidase-labeled secondary antibodies. Finally, the blots were detected using the enhanced chemiluminescence assay.

### MTS cell proliferation and colony formation assays

For the MTS cell proliferation assay, HCC cells (1000 cells/well) were seeded into the 96-well plates. After grown for 12 h, 10 μl MTS (0.2%)–PMS (0.092%; phenazine methosulfate, 20:1) solution was added to each well of the 96-well plates and incubated for 1.5 h at 37 °C. Cell viability was measured at 490 nm using a microplate reader.

For the colony formation assay, HCC cells (1000 cells/well) were seeded into the 6-well plates and then cultured for 15 days. Colonies were fixed with 4% paraformaldehyde for 15 min and stained with 0.5% crystal violet for 20 min at room temperature. Then, the colonies were pictured under a light microscope and the numbers of visible cell colonies were counted.

### Flow cytometry analysis of cell cycle and apoptosis

Cell cycle distribution was analyzed with a cell-cycle analysis kit (C-6031, US Everbright Inc). Briefly, HCC cells with different SLC25A20-expression levels were collected and fixed in 80% cold ethanol at −20 °C for 2 h. Then, 0.5 ml propidium iodide (PI) reagent was added to the cells and incubated for 20 min in the dark at 4 °C. Lastly, cell-cycle distribution in each cell group was evaluated by flow cytometry (Beckman, Fullerton, CA).

Cell apoptosis was determined with an Annexin V (FITC-conjugated)-apoptosis kit (F-6012, US Everbright Inc). Briefly, HCC cells with different SLC25A20-expression levels were collected and stained with 100 mL FITC-Annexin V and PI, and incubated for 15 min at room temperature in the dark. Lastly, cell apoptosis in each cell group was evaluated by flow cytometry (Beckman, Fullerton, CA).

### Wound-healing migration and transwell-invasion assays

The wound-healing assay was performed for the determination of cell migration. Briefly, HCC cells with different treatment suspended in serum-free medium were seeded into the 6-well plate. A scratch in the middle of the well was made when cells grown to 90% confluent. Photos of the wound region were taken at 0 h and 48 h after scratching. Relative wound closure in each group was calculated by image-J software.

For the determination of cell invasion, the 24-well transwell chamber coated with extracellular matrix was used. HCC cells suspended in serum-free medium were seeded into the upper chamber of the wells. After 48 h incubation at 37 °C, non-invasive cells on the upper surface were gently removed with a cotton-tipped swab and the penetrated cells were fixed with 4% paraformaldehyde and stained with 5% crystal violet. The number of invaded cells was counted under a light microscope.

### Immunohistochemistry (IHC) analysis

Paraffin-embedded tissue samples from patients with HCC or xenograft mouse tissues were cut into 4 μm sections. Tissue sections were deparaffinised in xylene and rehydrated in a series of graded alcohol, followed by blockage for endogenous peroxidase, and, antigen retrieval with hot citrate buffer (pH = 6) under pressure. Then, the sections were incubated overnight with specific primary antibodies listed in Supplementary Table 3 followed by detection with an IHC kit (Invitrogen). The staining intensity was evaluated by two independent pathologists who were blinded to the patients’ information on a scale of 1–12 according to the percentage of positive staining cells and their staining intensities.

### In vivo animal assays

For xenograft tumor growth assay, 4-week-old male BALB/c nude mice were randomized and divided into two groups (6 mice/group). A total of 1×10^7^ HLF cells with different SLC25A20-expression levels were subcutaneously injected into the right dorsal flank of the mice. Tumor volume was measured every week for 4 weeks.

For the metastasis model, 4-week-old male BALB/c nude mice were randomized and divided into two groups (6 mice/group). A total of 5×10^6^ HLF cells with different SLC25A20-expression levels were intravenously injected through the tail vein of the mice. Animals were killed at 6 weeks after injection and metastatic nodules formed in the lungs were determined by hematoxylin and eosin (HE) staining assay.

All animal experimental procedures were approved by the Animal Ethics Committee of the Fourth Military Medical University.

### TUNEL assay

TUNEL assay was applied with an in situ cell death detection kit (Roche, 11684795910) in xenograft tumor tissues from nude mice following the manufacturer’s protocol. Results were evaluated under a fluorescence microscope by calculating the percentage of TUNEL-positive cells in each group.

### Fatty-Acid β-oxidation detection

HCC cells (2 × 10^6^ cells/well) with different levels of SLC25A20 were cultured in 6-well plate. Then, 2 μCi [9, 10(n)-3H] oleic acid (Amersham Pharmacia Biotech, Italy) in 1 ml of HBSS containing 0.5 mg fatty-acid-free BSA was added and cultured for 12 h. The aqueous phase containing ^3^H_2_O was collected and extracted by using chloroform/ methanol (2:1 v/v). Later, scintillation solution was added and radioactivity was detected with a L6500 scintillation counter (Beckman Coulter, Brea, CA).

### Quantification of free fatty acid, triglyceride, and phospholipids

HCC cells with different levels of SLC25A20 were lysed in RIPA buffer at room temperature for 30 min. Then, lipids extraction was prepared using the chloroform/methanol (2:1). Later, the levels of free fatty acid, triglyceride, and phospholipids were measured using the EnzyChrom^TM^ free fatty acid, triglyceride, and phospholipid kits (Bioassay Systems, Hayward, CA, USA) following their manufacturers’ instructions, respectively.

### Statistical analysis

Experiments were performed independently three times, where appropriate. Experimental data are presented as mean ±standard error of the mean (SEM). All statistical analysis was performed using SPSS 17.0 software (SPSS, Chicago, IL). Kaplan–Meier method was used for survival analysis. Student’s *t*-test or One-way ANOVA was used for comparisons between two or multiple groups where appropriate. Differences were considered statistically significant when *P* value was less than 0.05.

## Results

### SLC25A20 is aberrantly down-regulated and its down-regulation is correlated with poor prognosis in HCC

**Fig. 1:**
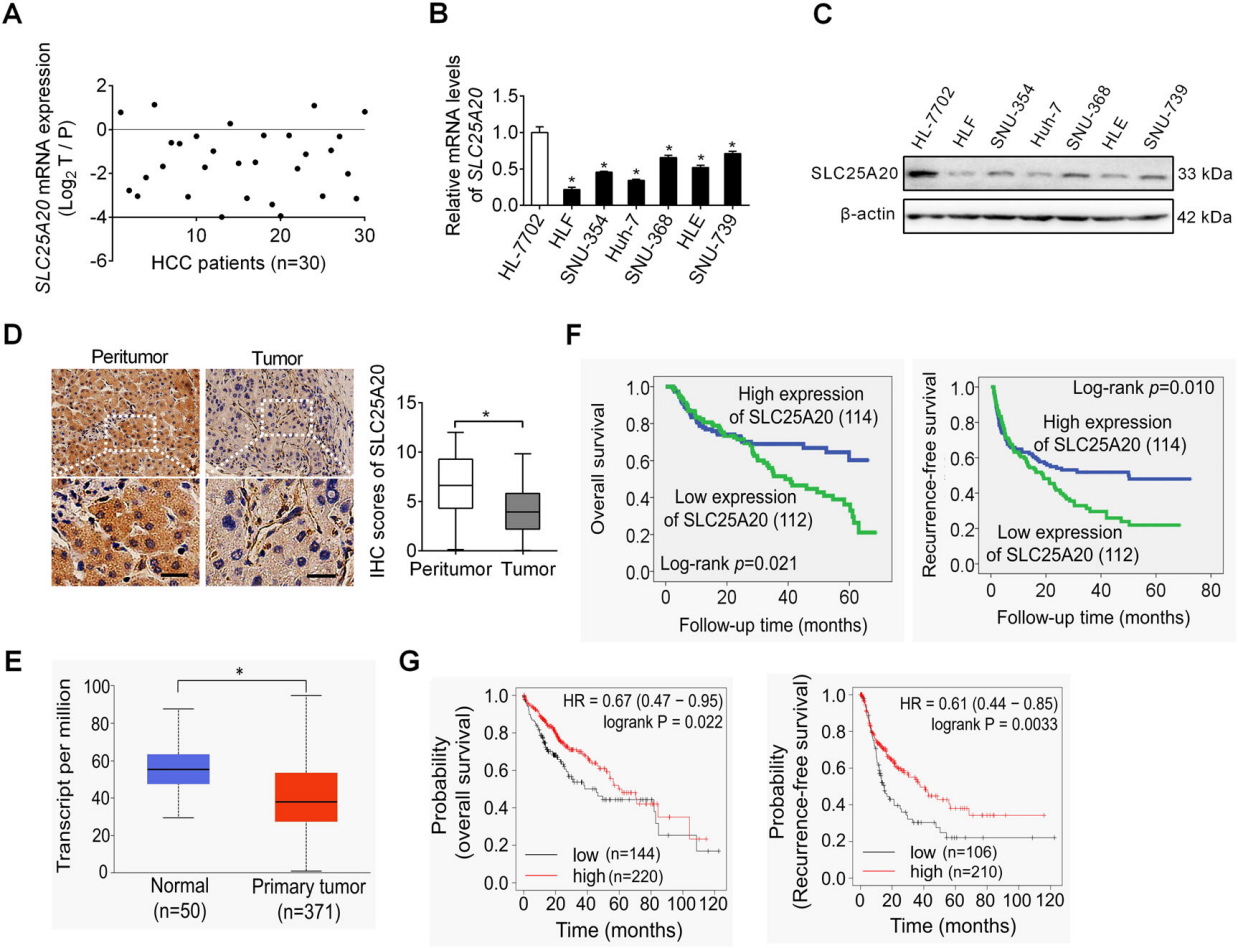
SLC25A20 is aberrantly down-regulated and its down-regulation is correlated with poor prognosis in HCC. **A** SLC25A20 expression was evaluated in 30-paired tumor and peritumor tissues from HCC patients using quantitative real-time PCR analysis. (T, tumor; P, peritumor). **B**, **C** SLC25A20 expression was determined in six HCC cell lines and one hepatocyte by using qRT–PCR and Western blot analysis. **D** Protein-expression levels of SLC25A20 were determined in 226-paired tumor and peritumor tissues of HCC using immunohistochemical (IHC) staining analysis. Scale bar, 20 μm. **E** Bioinformatics analysis for SLC25A20 expression at mRNA based on the online web portal UALCAN (http://ualcan.path.uab.edu). **F** Prognostic significance of SLC25A20 with Kaplan–Meier curves analysis based on the IHC analysis results in 226 patients with HCC. **G** Prognostic significance of SLC25A20 with Kaplan–Meier curves analysis based on the online web portal Kaplan–Meier Plotter in patients with HCC.

The expression of SLC25A20 was, firstly, analyzed in 30-paired tumors and the corresponding adjacent tissues of HCC using qRT–PCR analysis. Our results showed that SLC25A20 expression was significantly down-regulated in HCC tissues compared with paired normal tissues (Fig. 1A). In keeping with the results from tissue samples, SLC25A20 expression was also significantly lower in the six HCC cell lines when compared with an immortalized-hepatic-epithelial cell line HL-7702 (Fig. 1B, C). To determine whether the protein-expression level of SLC25A20 is also decreased in HCC tissues, we performed immunohistochemical analysis to further analyze the expression of SLC25A20 in the paired tumor and the peritumor tissues from another 226 patients with HCC. As shown in Fig. 1D, the protein-expression levels of SLC25A20 were commonly down-regulated in HCC tumor tissues compared with peritumor tissues. In consistency with our findings, bioinformatic analysis based on the online web portal UALCAN^11^ also revealed a significant down-regulation of SLC25A20 in liver tumor tissues when compared to normal liver tissues (Fig. 1E). Additionally, we found that the expression level of SLC25A20 was positively correlated with the maximum diameters of tumor, tumor-nodes-metastases (TNM) stage, and the incidence of portal vein tumor thrombosis (PVTT) of patients with HCC (Supplementary Table 1), implying a crucial role for SLC20A20 in HCC progression. Kaplan–Meier survival analysis further indicated that patients with HCC, with low expression levels of SLC25A20, had a significant shorter overall survival (OS) and recurrence-free survival (RFS) compared with those patients with high SLC25A20 expression levels (Fig. 1F), which was validated by bioinformatic analysis based on the online web portal Kaplan–Meier Plotter^12^ (Fig. 1G). These findings collectively indicate that SLC25A20 is down-regulated in HCC, which predicts a poor prognosis for patients with HCC. Given that hypoxia is common during tumor progression, we thus explored whether the down-regulation of SLC25A20 in HCC is associated with hypoxia. We found no significant change of SLC25A20 expression upon treatment with hypoxia (1% or 3% O_2_) in HLF and Huh-7 cells (Fig S1A), indicating that the down-regulation of SLC25A20 in HCC cells may not be caused by hypoxia.

### Forced expression of SLC25A20 suppressed HCC growth by inducing G1–S cell-cycle arrest and cell apoptosis

**Fig. 2:**
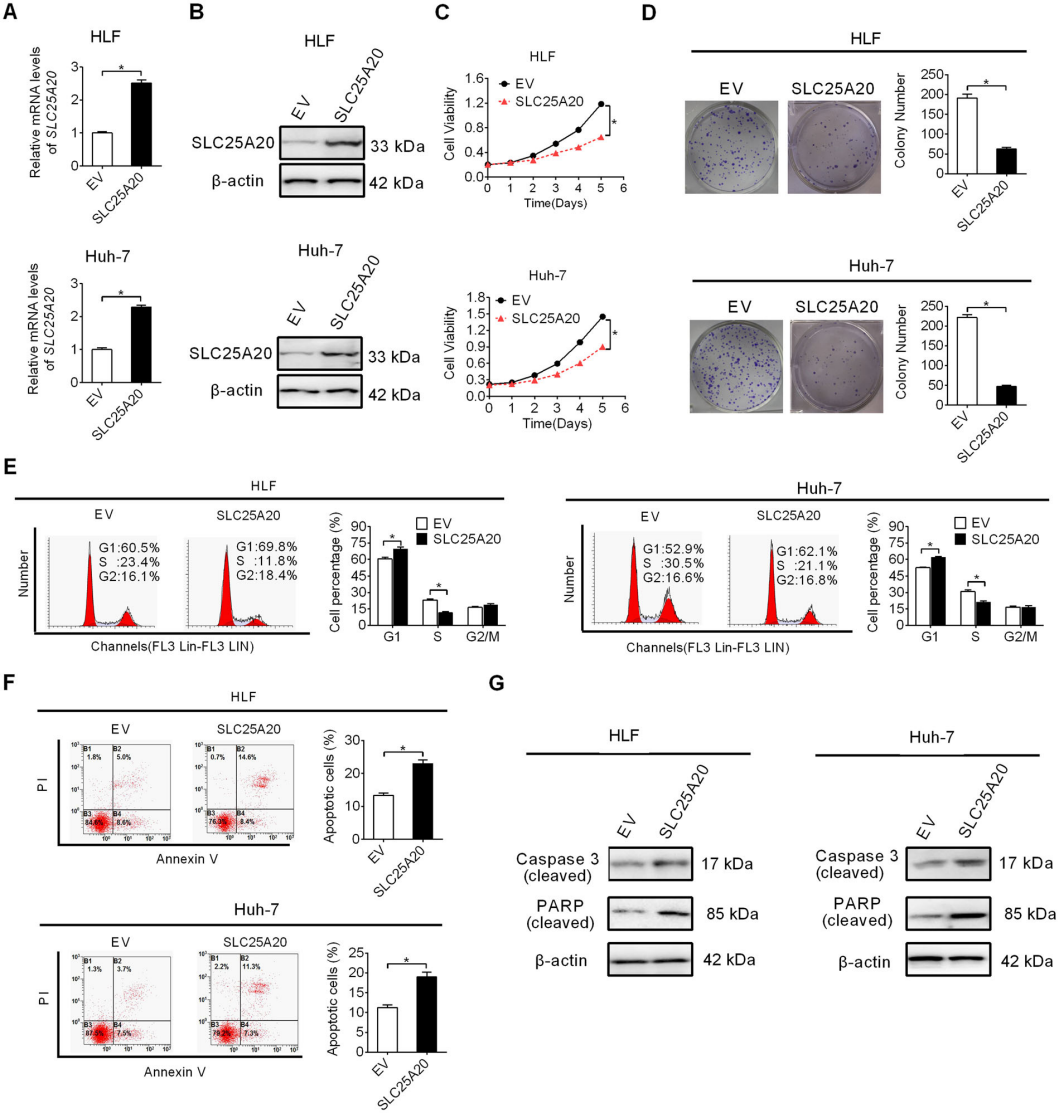
Forced expression of SLC25A20 suppressed HCC growth by inducing G1–S cell-cycle arrest and cell apoptosis. **A**, **B** SLC25A20 overexpression was confirmed by qRT–PCR and Western blot analysis in HLF and Huh-7 cells with treatment as indicated. (SLC25A20, expression vector encoding SLC25A20; EV, empty vector). **C**, **D** Cell growth was determined by MTS cell viability and colony formation assays in HLF and Huh-7 cells with treatment as indicated. **E**, **F** Cell cycle distribution and cell apoptosis were determined by flow cytometry analysis in HLF and Huh-7 cells with treatment as indicated. **G** Western blot analysis for levels of cleaved caspase-3 and cleaved PARP in HLF and Huh-7 cells with treatment as indicated.

To explore the effect of decreased expression of SLC25A20 on HCC cells, we overexpressed SLC25A20 in HLF and Huh-7 cells, which have relatively low SLC25A20 expression levels (shown in Fig. 1B, C). Significant overexpression of SLC25A20 was firstly confirmed by qRT–PCR and Western blot analysis (Fig. 2A, B). Then, we found that forced expression of SLC25A20 markedly suppressed cell proliferation in HLF and Huh-7 cells, as evidenced by an MTS cell viability and the colony formation assays (Fig. 2C, D). Given that both reduced cell-cycle progression and enhanced apoptosis could contribute to decreased cell proliferation, the effects of SLC25A20 overexpression on cell-cycle distribution and apoptosis were thus determined to explore the molecular mechanism by which SLC25A20 overexpression decreased HCC cell proliferation. As shown in Fig. 2E, SLC25A20 overexpression led to a significant arrest of G1-to-S cell cycle in HLF and Huh-7 cells. Cell apoptosis analysis revealed significantly more apoptotic cells in SLC25A20-overexpression HLF and Huh-7 cells than in their control cells (Fig. 2F). Consistently with this, the cleavage of caspase-3 and PARP were also significantly induced upon SLC25A20 overexpression in HLF and Huh-7 cells (Fig. 2G). These data suggest that SLC25A20 suppresses HCC growth through inducing cell-cycle arrest from G1-to-S and cell apoptosis.

### Forced expression of SLC25A20 inhibited HCC cell invasion and migration through suppression of epithelial–mesenchymal transition (EMT)

**Fig. 3:**
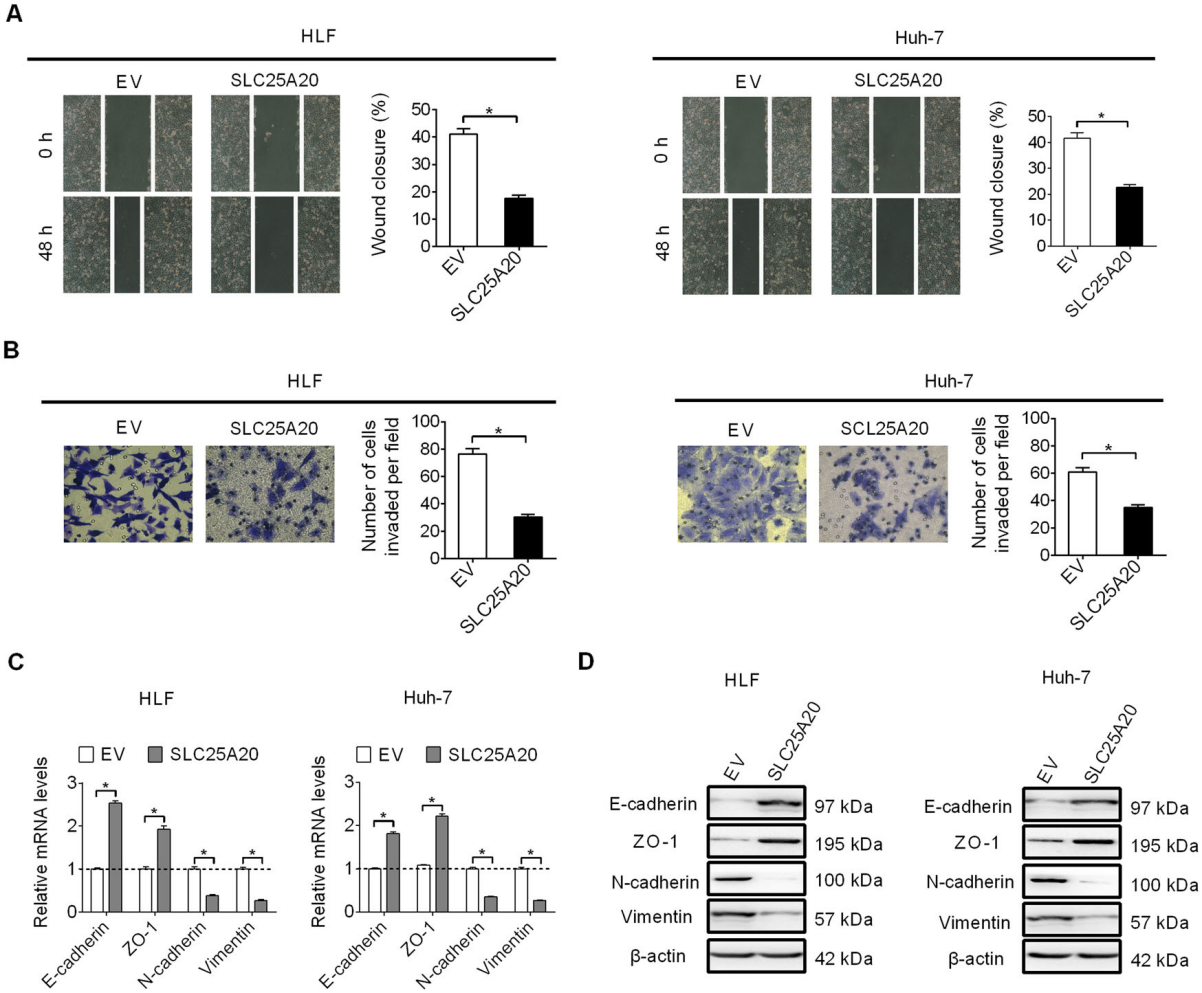
Forced expression of SLC25A20 inhibited HCC cell invasion and migration through suppression of epithelial–mesenchymal transition (EMT). **A** Cell migration ability was assessed using wound-healing assay in HLF and Huh-7 cells with treatment as indicated (SLC25A20, expression vector encoding SLC25A20; EV, empty vector). **B** Cell invasion ability was assessed by transwell matrigel-invasion assay in HLF and Huh-7 cells with treatment as indicated. **C**, **D** The expressions of EMT markers were determined by quantitative real-time PCR and Western blot analysis in HLF and Huh-7 cells with treatment as indicated.

We next examined the effect of SLC25A20 overexpression on HCC cell migration and invasion by using the wound-healing migration and the transwell-invasion assays, respectively. The wound-healing migration assay showed that SLC25A20 overexpression markedly suppressed wound closure efficiency of HLF and Huh-7 cells (Fig. 3A). Matrigel-invasion assay also showed that SLC25A20 overexpression promoted the invasion abilities of HLF and Huh-7 cells (Fig. 3B). Given that epithelial–mesenchymal transition (EMT) plays a critical role in the promotion of metastasis in human cancers^13^, the effect of SLC25A20 overexpression on the expressions of key markers in EMT was evaluated by qRT–PCR and Western blot analysis. As shown in Fig. 3C, D, SLC25A20 overexpression significantly increased the expressions of epithelial markers (E-cadherin and ZO-1), while it decreased the expressions of mesenchymal markers (N-cadherin and Vimentin), indicating that SLC25A20 overexpression may suppress HCC cell migration and invasion through repression of EMT.

### Forced expression of SLC25A20 suppressed HCC growth and metastasis in vivo

**Fig. 4:**
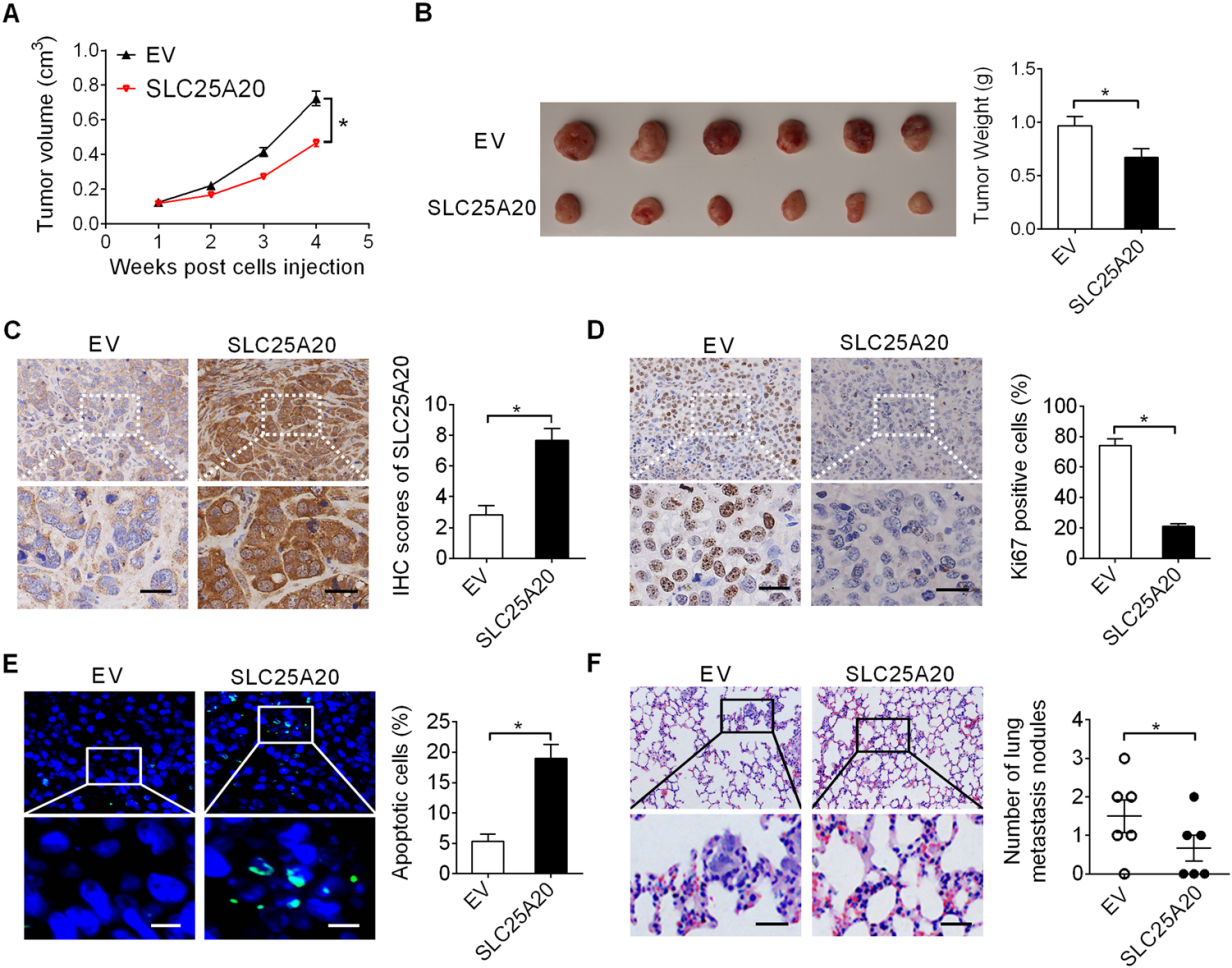
Forced expression of SLC25A20 suppressed HCC growth and metastasis in vivo. **A** Tumor-growth curves of xenografts established from HLF cells transfected with SLC25A20 expression vector (SLC25A20) or empty vector (EV). **B** The weights of tumors dissected from the nude mice at termination of the experiment were compared. **C**, **D** The expression levels of SLC25A20 and Ki-67 were determined by IHC staining analysis in xenografts developed from HLF cells transfected with SLC25A20 expression vector or empty vector. Scale bars, 10 μm. **E** TUNEL staining in xenografts developed from HLF cells transfected with SLC25A20 expression vector or empty vector. Scale bars, 5μm. **F** Incidences of lung metastases were determined in the two groups with different levels of SLC25A20 expression. Scale bars, 10 μm.

We further investigated the effect of SLC25A20 overexpression on HCC growth and metastasis in vivo. HLF cells with SLC25A20 stably overexpressed or control cells (Fig S1B, C) were subcutaneously injected into the right flanks of nude mice. As shown in Fig. 4A, the growth rates of tumors in SLC25A20 overexpression (SLC25A20) group were much slower than those in the empty vector (EV) group. Consistently with this, the net wet weights of tumors dissected from the nude mice in SLC25A20 group at the termination of the experiment were also significantly lower compared with those from EV group (Fig. 4B), suggesting that SLC25A20 overexpression suppressed the growth of HLF cells in vivo. Immunohistochemical staining showed a significant increase of SLC25A20 expression in xenografts developed from SLC25A20 overexpression (SLC25A20) group compared with the empty vector (EV) group (Fig. 4C), suggesting that the tumor-suppressive effect was exerted by SLC25A20 overexpression. Additionally, Ki-67 and TUNEL staining assays indicated fewer proliferating and more apoptotic cells in xenografts developed from SLC25A20 overexpression (SLC25A20) group compared with the empty vector (EV) group (Fig. 4D, E), which is consistent with the in vitro findings that SLC25A20 overexpression suppressed HCC cell proliferation but induced cell apoptosis. Furthermore, significantly fewer metastatic nodules in the lungs from SLC25A20 overexpression (SLC25A20) group were observed when compared with the empty vector (EV) group, as revealed by the in vivo tail vein metastasis assay (Fig. 4F).

### Knockdown of SLC25A20 enhanced HCC cell growth and metastasis

**Fig. 5:**
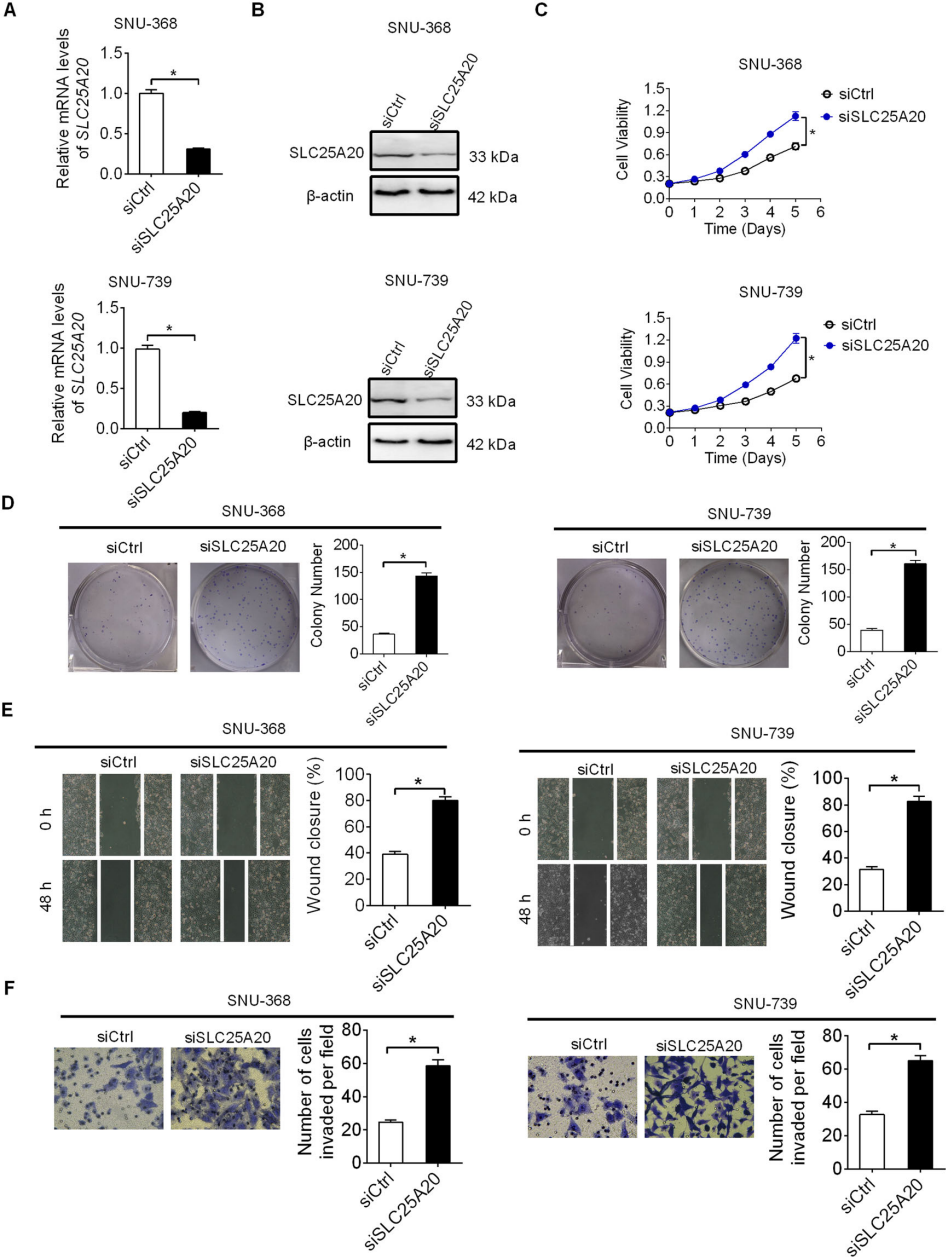
Knockdown of SLC25A20 enhanced HCC cell growth and metastasis. **A**, **B** Knockdown of SLC25A20 was confirmed by qRT–PCR and Western blot analysis in SNU-368 and SNU-739 cells with treatment as indicated. (siSLC25A20, siRNA against SLC25A20; siCtrl, control siRNA). **C**, **D** MTS cell viability and colony formation assays were applied in SNU-368 and SNU-739 cells with treatment as indicated. **E**, **F** Wound-healing and matrigel-invasion assays were used for determination of cell metastasis in SNU-368 and SNU-739 cells with treatment as indicated.

To provide further support for the anti-tumorigenic effect of SLC25A20, we knocked-down SLC25A20 expression in SNU-368 and SNU-739 cells with relative high SLC25A20-expression levels, as shown in Fig. 1B, C. SLC25 A20 knockdown in SNU-368 and SNU-739 cells was confirmed by qRT–PCR and Western blot analysis (Fig. 5A, B). Significantly elevated cell viability and colony formation abilities were observed when SLC25A20 was knocked-down in SNU-368 and SNU-739 cells (Fig. 5C, D). Additionally, knockdown of SLC25A20 also remarkably increased the migration and invasion abilities of SNU-368 and SNU-739 cells (Fig. 5E, F).

### Decreased SLC25A20 expression is mainly mediated by frequent overexpression of miR-132-3p in HCC cells

DNA methylation and miRNAs are both critical regulators of gene expression. To explore the upstream molecular mechanism leading to the down-regulation of SLC25A20 in HCC, we firstly analyzed the promoter methylation status of SLC25A20 in HCC by using the online web portal UALCAN. However, no significant change of the promoter methylation status of SLC25A20 was observed in primary tumor tissues when compared with normal liver tissues (Fig S2A). Then, to investigate the potential microRNAs involved in the down-regulation of SLC25A20 in HCC, microRNA Data Integration Portal (mirDIP) analysis was applied to predict potential miRNA targeting SLC25A20. Our results indicated that, among the top ten predicted miRNAs targeting SLC25A20 (Fig S2B), only transfection of miR-132-3p significantly reduced SLC25A20 expression in SNU-368 and SNU-739 cells (Fig. S2C, D). Expectedly, a significant overexpression of miR-132-3p (Fig S2E) and a negative correlation between the expressions of miR-132-3p and SLC25A20 (Fig. S2F; *r* = −0.493, *p* = 0.012) were observed in tumor tissues from patients with HCC. These results were further supported by the bioinformatics analysis based on the ENCORI database^14^, showing the overexpression of miR-132-3p in HCC, which predicts poor survival for patients with HCC, as well as the negative correlation between the expressions of miR-132-3p and SLC25A20 (Fig S2G–I). Moreover, miR-132-3p transfection greatly reversed SLC25A20-overexpression–suppressed-HCC-growth-and-metastasis, as evidenced by MTS cell viability, colony formation, wound-healing migration, and transwell-invasion assays (Fig. S3A–E).

### SLC25A20 down-regulation promoted HCC growth and metastasis through suppression of fatty-acid oxidation

**Fig. 6:**
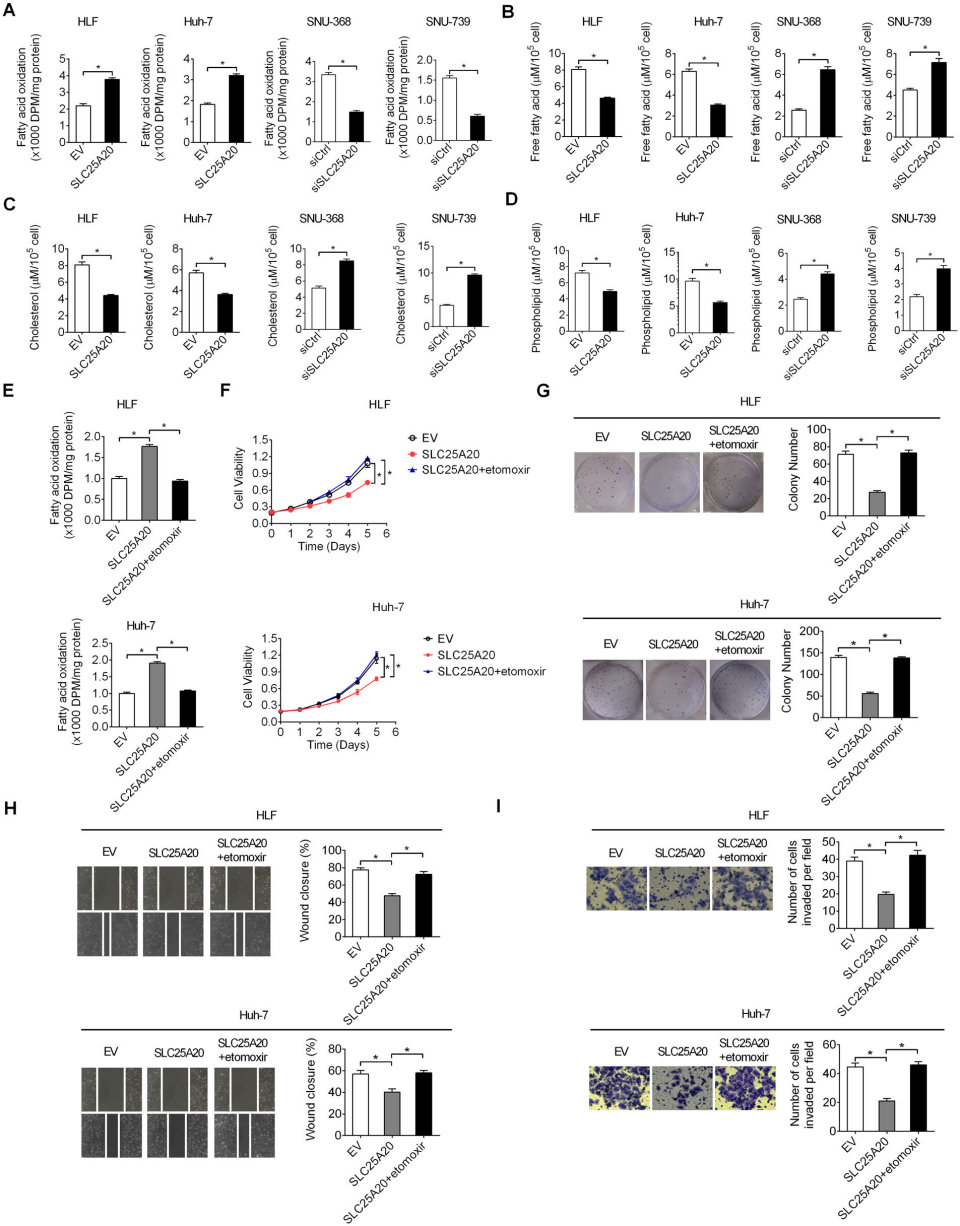
SLC25A20 down-regulation promoted HCC growth and metastasis through suppression of fatty-acid oxidation. **A** ^3^H-labeled oleic acid was used to access the ability of fatty-acid oxidation in HCC cells with treatment as indicated. **B**, **D** Intracellular levels of free fatty acid (FFA), triglyceride (TAG), and phospholipids (PL) were determined in HCC cells with treatment as indicated. **E** Fatty-acid oxidation was determined in HCC cells with treatment as indicated. **F**, **G** Cell growth ability was determined by MTS cell viability and colony formation assays in HCC cells with treatment as indicated. **H**, **I** Cell metastasis was determined by wound-healing and matrigel-invasion assays in HCC cells with treatment as indicated.

SLC25A20 is a mitochondrial-membrane–carrier protein involved in the transport of acylcarnitines into mitochondrial matrix for oxidation. To determine the mechanism by which SLC25A20 exerts its anti-tumorigenic roles in HCC, the effect of SLC25A20 overexpression or knockdown on fatty-acid oxidation was determined. As shown in Fig. 6A, SLC25A20 overexpression significantly increased fatty-acid oxidation in HLF and Huh-7 cells, while SLC25A20 knockdown suppressed fatty-acid oxidation in SNU-368 and SNU-739 cells. SLC25A20 overexpression resulted in a significant decrease of intracellular lipid content, including free fatty acid (FFA), triglyceride (TAG), and phospholipids (PL), while knockdown of SLC25A20 caused lipid accumulation in HCC cells (Fig. 6B–D). Previous studies have demonstrated that Acyl-CoA dehydrogenase medium-chain (ACADM), acyl-CoA dehydrogenase long-chain (ACADL), and patatin-like phospholipase domain containing 2 (PNPLA2) are three critical regulators in fatty-acid oxidation^15^. We then determined if ACADM, ACADL, and PNPLA2 were involved in SLC25A20-regulated fatty-acid oxidation in HCC cells. No significant changes of ACADM, ACADL, and PNPLA2 expressions were observed when SLC25A20 was overexpressed in HLF cells or knocked-down in SNU-368 cells, indicating that the regulation of fatty-acid oxidation by SLC25A20 is independent of ACADM, ACADL, and PNPLA2 in HCC cells (Fig S4). To further determine whether SLC25A20 suppressed HCC growth and metastasis through induction of fatty-acid oxidation, fatty-acid oxidation was inhibited by using etomoxir, an inhibitor of fatty-acid oxidation through targeting carnitine pamitoyltransferase-1 (CPT1). As shown in Fig. 6E, treatment with etomoxir significantly suppressed SLC25A20 overexpression-induced FAO. In addition, FAO inhibition by etomoxir treatment significantly reversed SLC25A20-overexpression–suppressed-HCC-cell-growth-and-metastasis (Fig. 6F–I), indicating that SLC25A20 suppressed HCC growth and metastasis through activation of fatty-acid oxidation.

## Discussion

In the present study, we have shown that SLC25A20 expression is frequently decreased in HCC cell lines and tumor tissues mainly due to the up-regulation of miR-132-3p. Clinical significance study by Pearson’s correlation indicated that the expression of SLC25A20 was negatively associated with the tumor size and vascular invasion in patients with HCC. Kaplan–Meier analysis also revealed SLC25A20 down-regulation as a prognostic factor for poor survival of patients with HCC. Consistently with this, a recent study using mass spectrometry (MS)-based proteomics analysis in tumor and adjacent non-tumor tissues from 159 patients with HCC has also revealed the down-regulation of SLC25A20 and its correlation with poor survival of patients with HCC^10^. In addition to SLC25A20, carnitine palmitoyltransferase 1 (CPT1) family, which contains 3 isoforms of CPT1A, CPT1B, and CPT1C, also localized in mitochondrial membrane and function as gate-keeper for the entry of long-chain fatty acids into mitochondria for oxidation^16^. During recent years, cumulative evidence has shown that CPT1 dysfunction was closely related to the development and progression of several metabolic disorders and human cancers^17^. Overexpression and oncogenic functions of CPT1A and CPT1C have been revealed in several types of human malignancies, including colorectal^18^, papillary thyroid^19^, and lung^20^ cancers.

Frequently decreased expression of SLC25A20 and its significant correlation with poor survival in patients with HCC imply that SLC25A20 may play an anti-tumorigenic role in the development and progression of HCC. In this connection, the function of SLC25A20 in HCC growth and metastasis was investigated both in vitro and in vivo. Forced expression of SLC25A20 significantly suppressed cell viability and colon formation in HLF and Huh-7 cells. In contrast, SLC25A20 knockdown remarkably enhanced cell viability and colon formation in SNU-368 and SNU-739 cells. The molecular mechanism underlying the tumor-growth-suppressive function of SLC25A20 was further demonstrated to be mediated by the simultaneous induction of G1–S cell-cycle arrest and cell apoptosis. Moreover, in concordance with the in vitro findings, subcutaneous-xenograft nude mice models also showed that SLC25A20 overexpression significantly attenuated tumorigenicity of HCC cells in vivo. Additionally, significantly fewer proliferating cells and more apoptotic cells were detected in the SLC25A20-overexpressing-subcutaneous-xenograft tumors compared with those in control.

In addition to the significance of SLC25A20 on tumor growth, the biological function of SLC25A20 on HCC cell migration and invasion was also explored both in vitro and in vivo. Forced expression of SLC25A20 in HLF and Huh-7 cells significantly suppressed their migration and invasion abilities. In addition, the molecular mechanism underlying the anti-invasive function of SLC25A20 was shown to be mediated by the promotion of epithelial–mesenchymal transition (EMT). In keeping with the in vitro results, significantly fewer metastatic nodules formed in the lungs as observed in SLC25A20 overexpression group compared with the empty vector control group, as revealed by the in vivo tail vein metastasis assay.

DNA methylation and miRNAs are both critical regulators of gene expression. Abnormal DNA methylation and miRNA expression have been shown to play important roles in the development and progression of cancer^21,22^. No significant change of the promoter methylation status of SLC25A20 was observed in primary tumor tissues when compared with normal liver tissues. MiR-132-3p is a highly conserved miRNA that has been shown to participate in the progression of different types of human cancers. In pancreatic cancer (PC), miR-132-3p was demonstrated to be significantly up-regulated compared with the normal and benign tissues, which contribute to the malignant progression of PC via regulating caspases-7^23^. Besides, overexpression of miR-132-3p has also been reported in cholangiocarcinoma and glioma endothelial tissues^24,25^. On the contrary, in colorectal and breast cancers^26,27^, miR-132-3p expression has been shown to be down-regulated and to play an anti-tumorigenic role in the progression of those cancers. In HCC, it has been reported that the level of miR-132-3p in the plasma was significantly increased in patients with HCC compared with the cancer-free controls^28^. Consistently with this, our present study also showed that miR-132-3p expression is significantly increased in tumor tissues of HCC compared with peritumor tissues. Moreover, we found that miR-132-3p was involved in the down-expression of SLC25A20 and, thus, its anti-tumorigenic functions in the suppression of HCC growth and metastasis follow. Nevertheless, we still can’t rule out the possibility that other gene expression regulators may also contribute to the down-regulation of SLC25A20 in HCC, which still needs further investigations.

SLC25A20 is a mitochondrial-membrane–carrier protein involved in the transport of acylcarnitines into mitochondrial matrix for acid oxidation. Consistent with our finding that SLC25A20 expression is down-regulated and plays anti-tumorigenic functions in HCC, a previous study using whole-genome microarray had also revealed that a series of metabolic enzymes involved in fatty-acid oxidation (FAO) were down-regulated in HCC^29^. Besides, another study on five different types of cancers has also demonstrated that the incorporation of fatty acid into oxidative pathway is reduced^30^. Our present study also showed that SLC25A20 down-regulation significantly suppressed fatty-acid oxidation in HCC cells, implying that cancer cells may suppress FAO to save more fatty acid in order to grow and divide. In addition, we demonstrated that suppressed fatty-acid oxidation was involved in the promotion of HCC growth and metastasis by SLC25A20 knockdown.

In summary, our present study for the first time demonstrates that SLC25A20 is frequently down-regulated in HCC, which predicts poor survival for patients with HCC. SLC25A20 plays a critical tumor-suppressive role by inhibiting both growth and metastasis of HCC. These findings suggest SLC25A20 as a potential prognostic factor and therapeutic target in the treatment of HCC.

## Supplementary information

supplementary figures and tables

author-contribution-form 1

author-contribution-form 2

aj-checklist
