# Supplementary material for: Down-regulation of SLC25A20 promotes hepatocellular carcinoma growth and metastasis through suppression of fatty-acid oxidation
Source: Cell Death Dis. 2021 Apr 6;12(4):361. doi: 10.1038/s41419-021-03648-1 (PMC8024385; doi:10.1038/s41419-021-03648-1)
Supplement: Supplementary file 1 — supplementary figures and tables [file 41419_2021_3648_MOESM1_ESM.docx]

**Supplemental information**

**Down-regulation of SLC25A20 promotes hepatocellular carcinoma growth and metastasis through suppression of fatty acid oxidation**

**Supplemental figures**

**
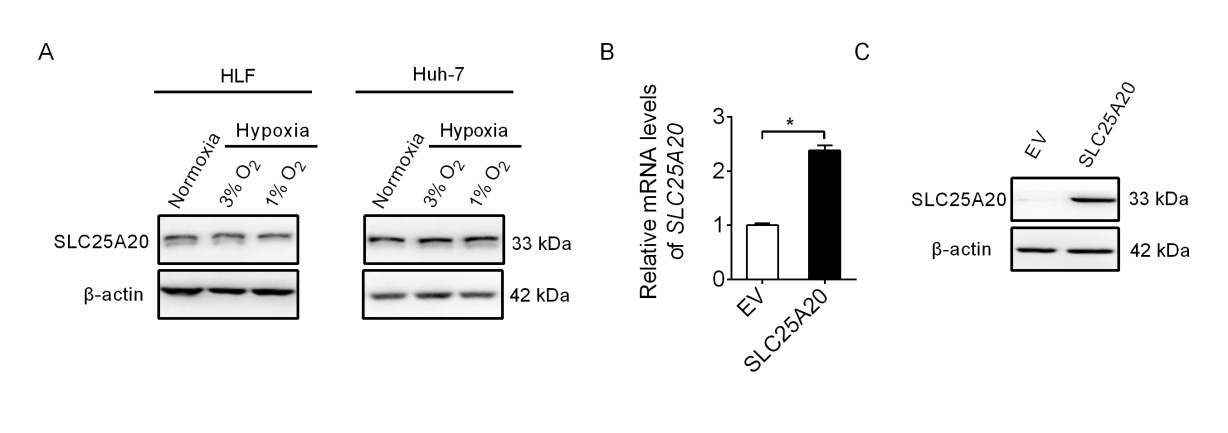
**

**Figure S1.** (A) The effect of hypoxia on the expression of SLC25A20 expression in HLF and Huh-7 cells. (B and C) Stable overexpression of SLC25A20 in HLF cells was determined by qRT-PCR **(A)** and Western blot (**B**) analysis.


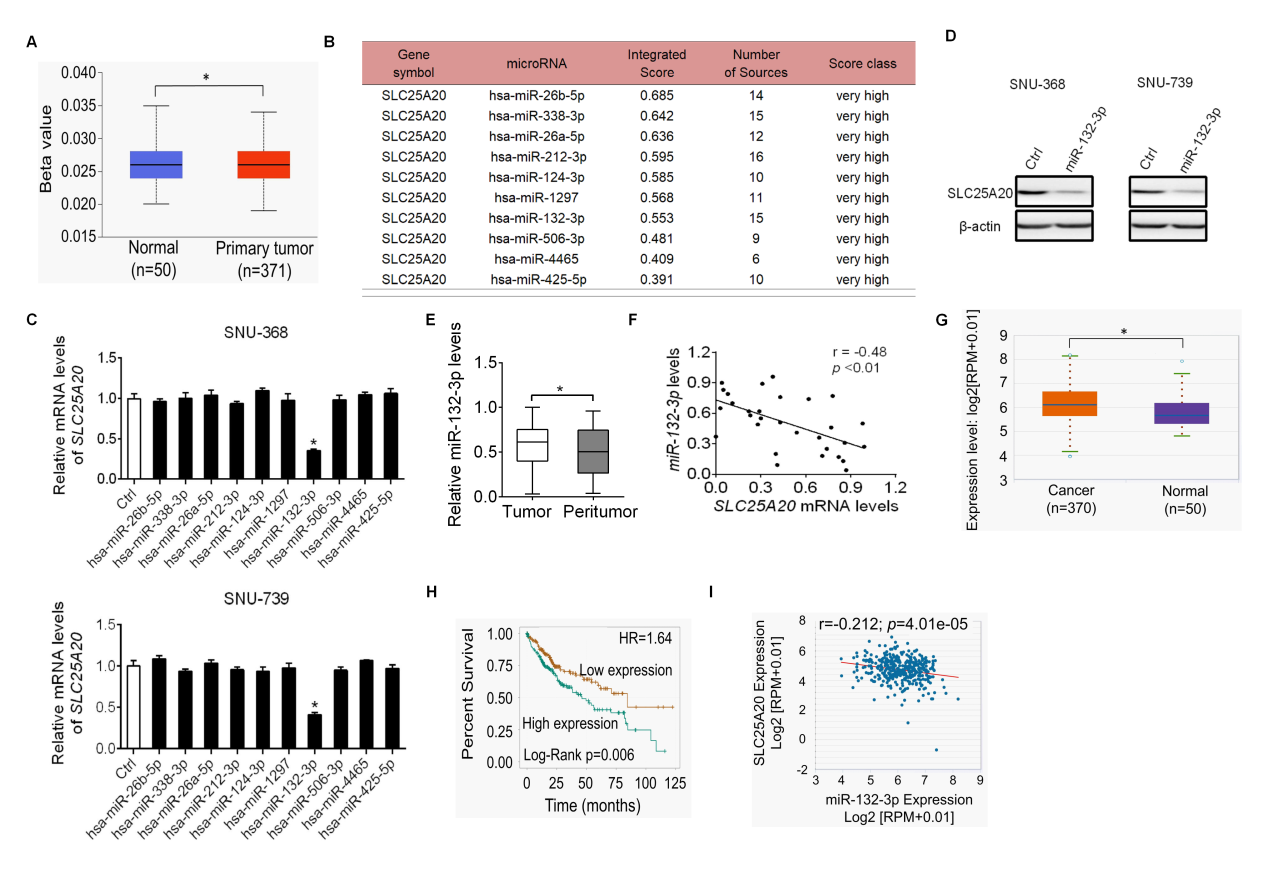


**Figure S2. Decreased SLC25A20 expression is mainly mediated by frequently overexpression of miR-132-3p in HCC cells. (A)** Promoter methylation status of SLC25A20 analysis based on the online web portal UALCAN was applied in HCC cells. **(B)** Top ten predicted miRNAs targeting SLC25A20 using the microRNA Data Integration Portal (mirDIP)-based target prediction^1^. **(C and D)** The expression of SLC25A20 was determined by qRT-PCR (C) and western blot (D) analysis in SNU-368 and SNU-739 cells after transfection with synthetic precursors of different miRNAs as indicated. **(E)** Expression of miR-132-3P was determined by qRT-PCR in tumor and peritumor tissues from patients with HCC. **(F)** Correlation between the expressions of SLC25A20 and miR-132-3p were determined in 30 HCC tumor tissues. **(G-I)** Bioinformatics analysis based on the ENCORI database ^2^for the expression of miR-132-3p (**G**) and its prognostic significance (**H**), as well as its correlation with SLC25A20 (**I**) in patients with HCC.


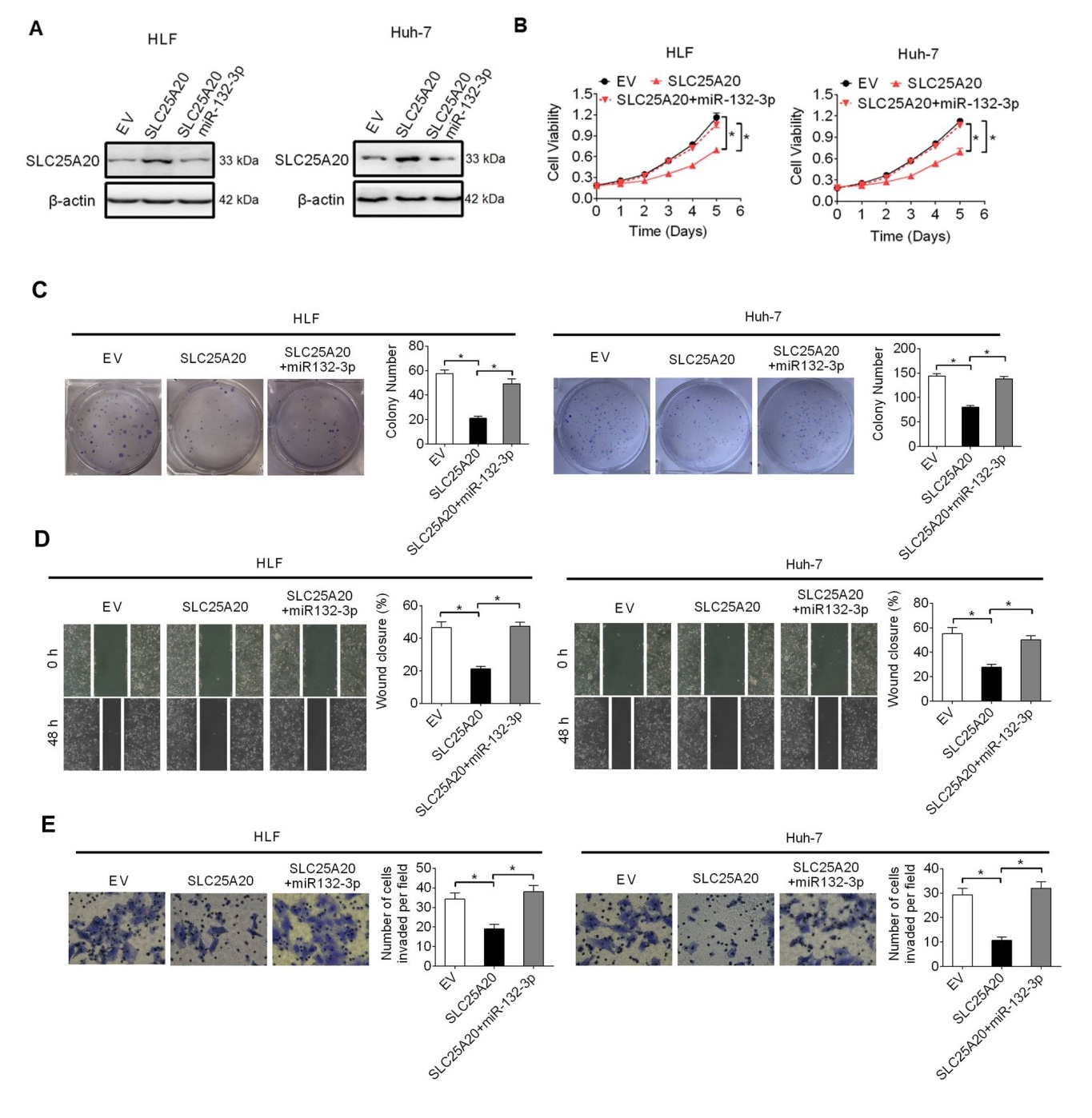


**Figure S3.** **miR-132-3p transfection greatly reversed SLC25A20 overexpression suppressed HCC growth and metastasis. (A)** SLC25A20 expression was determined by Western blot analysis in HLF and Huh-7 cells with different treatment as indicated. **(B and C)** Cell growth ability was determined by MTS cell viability and colony formation assays in HLF and Huh-7 cells with treatment as indicated. **(D and E)** Cell metastasis was determined by wound healing and matrigel invasion assays in HLF and Huh-7 cells with treatment as indicated.

**
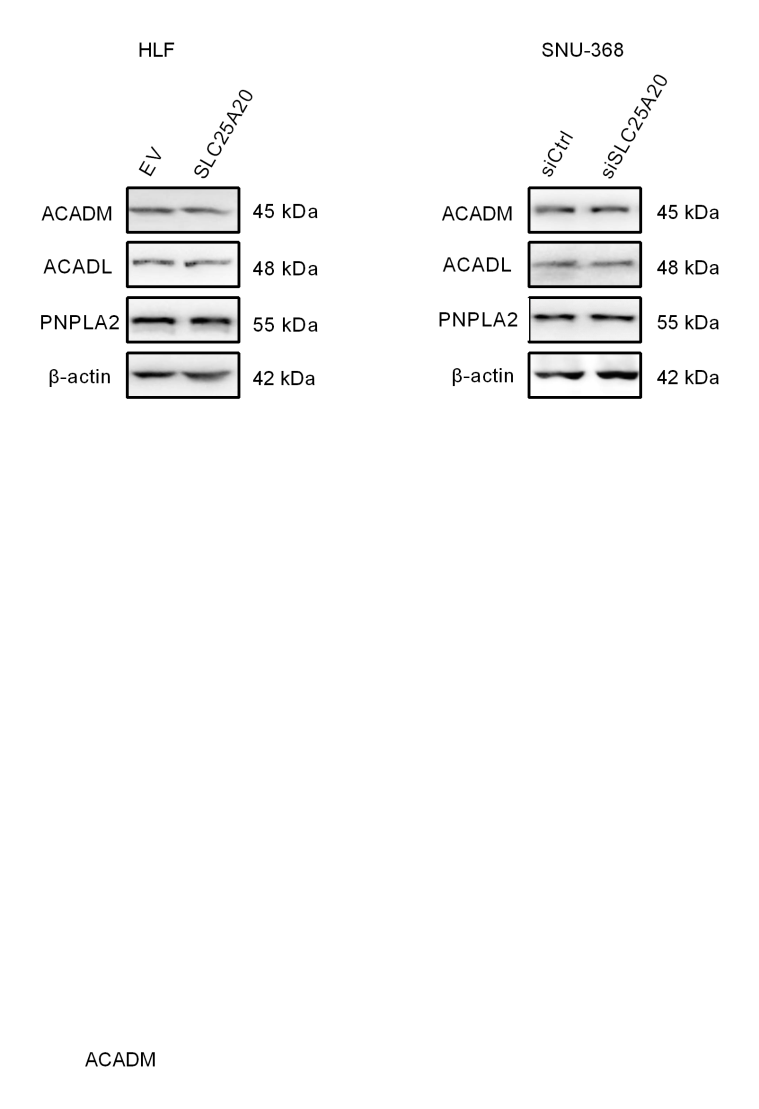
**

**Figure S4.** The effects of overexpression or knock-down of SLC25A20 on ACADM, ACADL and PNPLA2 expressions were determined by western blot analysis in HLF and SNU-368 cells.**Supplementary Tables**

**Table S1. Correlation between SLC25A20 expression level and clinicopathologic features in 226 HCC patients.**

| Variables | No. of cases (%) | TFB1M expression | | *P* value |
| --- | --- | --- | --- | --- |
|  |  | Low | High |  |
| All | 226 (100%) | 112 | 114 |  |
| Age |  |  |  |  |
| <55 | 95 ( %) | 44 | 51 | 0.42 |
| >=55 | 131 (%) | 68 | 63 |  |
| Gender |  |  |  |  |
| Female | 38 (%) | 21 | 17 | 0.48 |
| Male | 188 (%) | 91 | 97 |  |
| HBV |  |  |  |  |
| Negative | 27 (%) | 16 | 11 | 0.31 |
| Positive | 199 (%) | 96 | 103 |  |
| alpha-fetoprotein (ug/ml) |  |  |  |  |
| <200 | 124 (%) | 59 | 65 | 0.59 |
| >=200 | 102 (%) | 53 | 49 |  |
| Maximum diameter of lesion |  |  |  |  |
| <5 | 119 (%) | 69 | 50 | **0.01** |
| >=5 | 107 (%) | 43 | 64 |  |
| tumor-nodes-metastases stage |  |  |  |  |
| I+ II | 173 (%) | 94 | 79 | **0.01** |
| III+ IV | 53 (%) | 18 | 35 |  |
| Differentiation grade |  |  |  |  |
| I+ II | 75 (%) | 39 | 36 | 0.67 |
| III | 151 (%) | 73 | 78 |  |
| portal vein tumor thrombosis |  |  |  |  |
| No | 166 (%) | 90 | 76 | **0.02** |
| Yes | 60 (%) | 22 | 38 |  |
| Treatment |  |  |  |  |
| Hepatectomy | 164 (%) | 77 | 87 | 0.23 |
| Hepatectomy+ TACE | 62 (%) | 35 | 27 |  |

**Table S2.** Sequence of primers for qRT-PCR analysis

| *SLC25A20* | forward primer | GGGGTCACTCCCATGTTTG |
| --- | --- | --- |
|  | reverse primer | TGTGGTGAATACGCCAGATAAC |
| *E-cadherin* | forward primer | GGCCCAGGAGCTGACAAAC |
|  | reverse primer | GTGGATGGCAAAGTGGTGTC |
| *Z0-1* | forward primer | CAACATACAGTGACGCTTCACA |
|  | reverse primer | CACTATTGACGTTTCCCCACTC |
| *N-cadherin* | forward primer | CACTGCTCAGGACCCAGAT |
|  | reverse primer | TAAGCCGAGTGATGGTCC |
| *Vimentin* | forward primer | TCGTTTCGAGGTTTTCGCGTTAGAGAC |
|  | reverse primer | CGACTAAAACTC GACCGACTCGCGA |
| miR-132-3p | forward primer | GCGCGCGTAACAGTCTACAGC |
|  | reverse primer | GTCGTATCCAGTGCAGGGTCC |
| U6 | forward primer | CTCGCTTCGGCAGCACA |
|  | reverse primer | AACGCTTCACGAATTTGCGT |
| *β-actin* | forward primer | GGCTGTATTCCCCTCCATCG |
|  | reverse primer | CCAGTTGGTAACAATGCCATGT |

**Table S3.** Primary antibodies used in this study.

| **Antibody** | **Company (Cat. No.)** | **Working dilutions** |
| --- | --- | --- |
| SLC25A20 | abcam (ab244436) | WB: 1/500; IHC:1/300 |
| E-cadherin | Proteintech (20874-1-AP) | WB: 1/1000 |
| Z0-1 | Proteintech (21773-1-AP) | WB: 1/1000 |
| N-cadherin | Proteintech (22018-1-AP) | WB: 1/1000 |
| Vimentin | abcam (ab8978) | WB: 1/1000 |
| Ki-67 | abcam (ab15580) | IHC:1/300 |
| Caspase 3 | Proteintech (19677-1-AP) | WB: 1/1000 |
| PARP | Proteintech (13371-1-AP) | WB: 1/1000 |
| ACADM | Proteintech (55210-1-AP) | WB: 1/1000 |
| ACADL | Proteintech (17526-1-AP) | WB: 1/1000 |
| PNPLA2 | Proteintech (55190-1-AP) | WB: 1/500 |
| β-actin | Proteintech (20536-1-AP) | WB: 1/1000 |

**Reference**

1. Tokar T, Pastrello C, Rossos AEM, Abovsky M, Hauschild AC, Tsay M*, et al.* mirDIP 4.1-integrative database of human microRNA target predictions. *Nucleic acids research* 2018, **46**(D1)**:** D360-D370.

2. Li JH, Liu S, Zhou H, Qu LH, Yang JH. starBase v2.0: decoding miRNA-ceRNA, miRNA-ncRNA and protein-RNA interaction networks from large-scale CLIP-Seq data. *Nucleic acids research* 2014, **42**(Database issue)**:** D92-97.
