# Supplementary figures and images for: Down-regulation of SLC25A20 promotes hepatocellular carcinoma growth and metastasis through suppression of fatty-acid oxidation
Source: Cell Death Dis. 2021 Apr 6;12(4):361. doi: 10.1038/s41419-021-03648-1 (PMC8024385; doi:10.1038/s41419-021-03648-1)

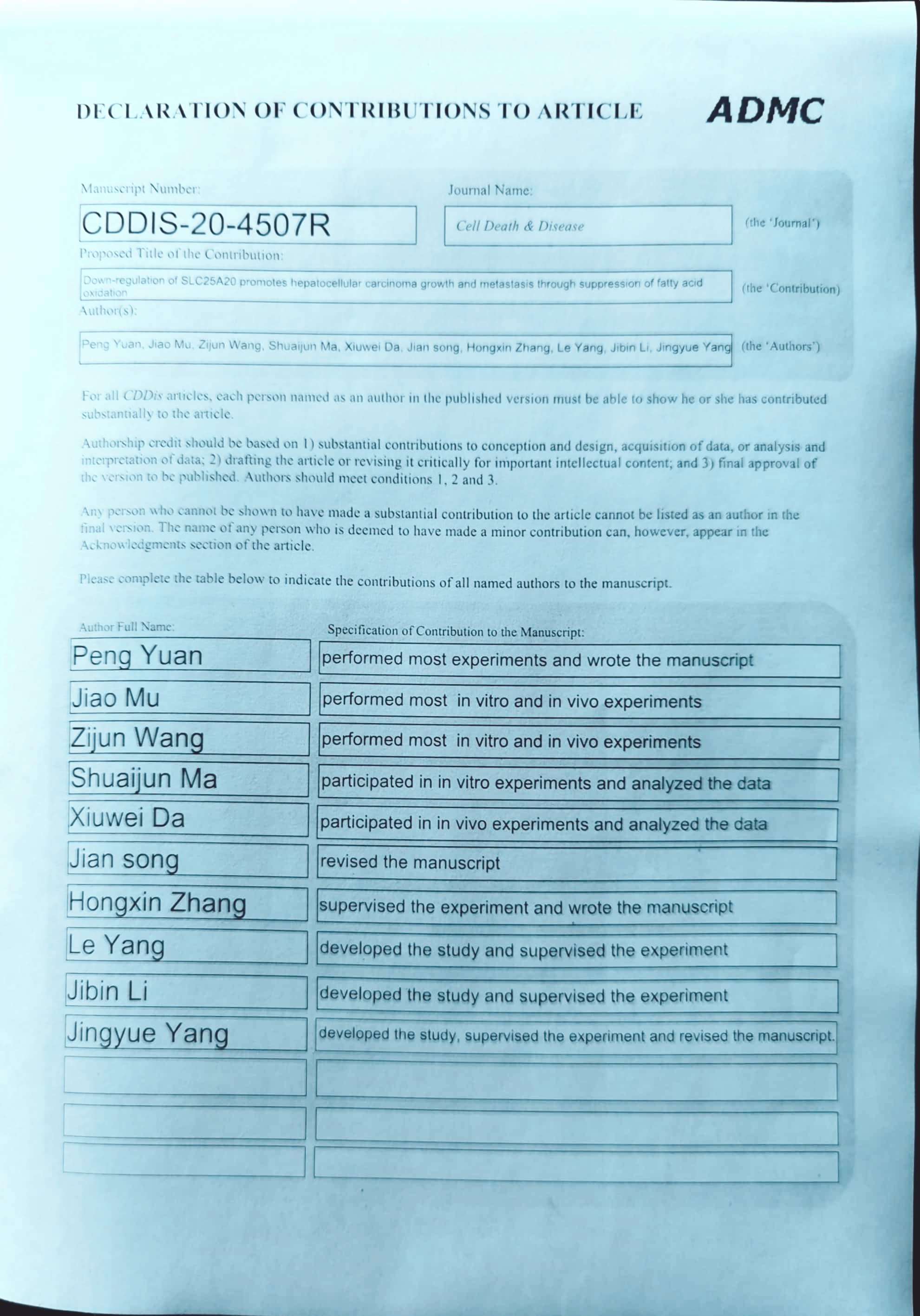

Supplement: Supplementary file 2 — author-contribution-form 1 [file 41419_2021_3648_MOESM2_ESM.jpg]

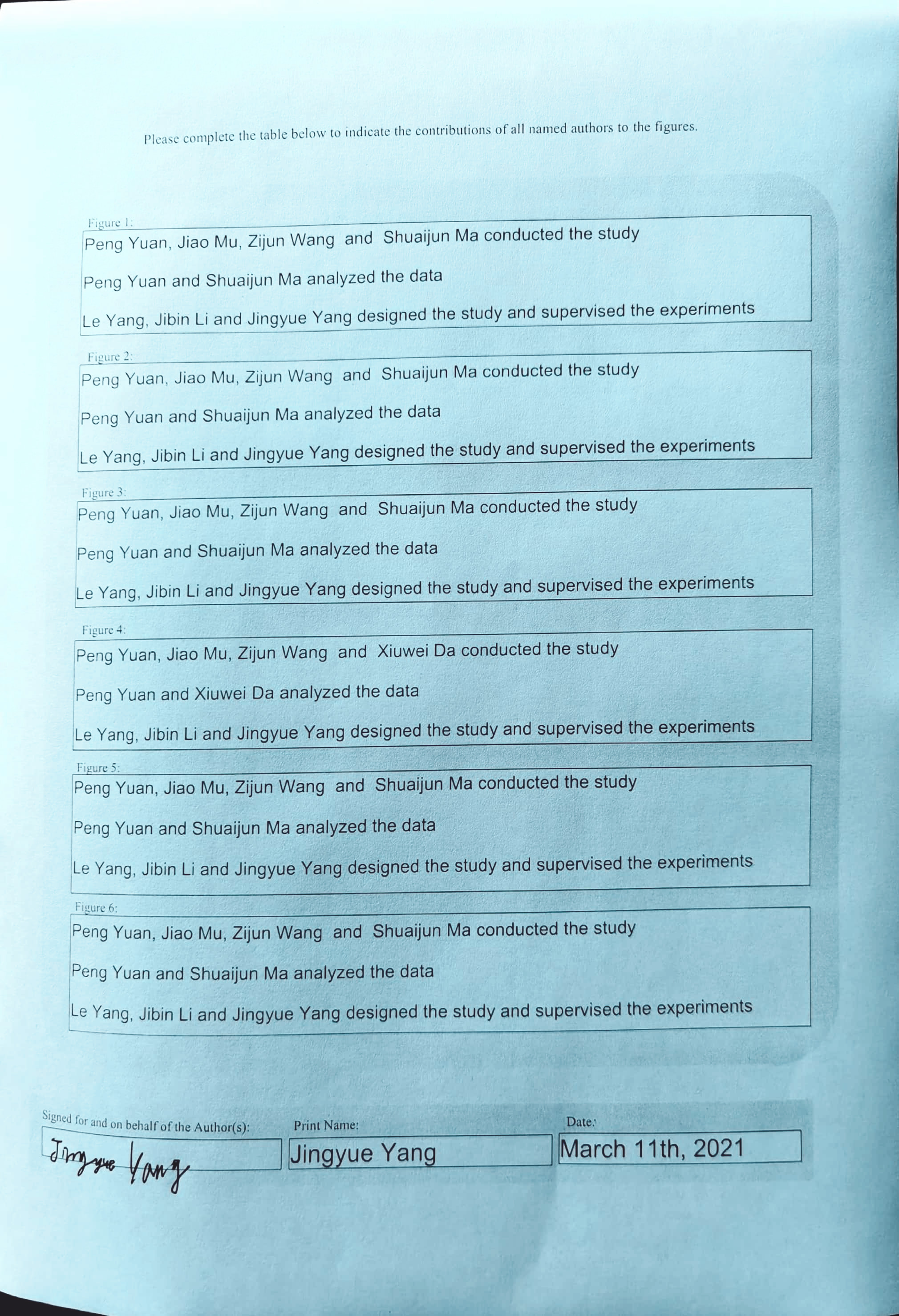

Supplement: Supplementary file 3 — author-contribution-form 2 [file 41419_2021_3648_MOESM3_ESM.jpg]
